# Extracting health-related causality from twitter messages using natural language processing

**DOI:** 10.1186/s12911-019-0785-0

**Published:** 2019-04-04

**Authors:** Son Doan, Elly W. Yang, Sameer S. Tilak, Peter W. Li, Daniel S. Zisook, Manabu Torii

**Affiliations:** Medical Informatics, Kaiser Permanente Southern California, San Diego, CA 92130 USA

**Keywords:** Twitter, Causality, Causal relationships, Cause-effect, Natural language processing (NLP)

## Abstract

**Background:**

Twitter messages (tweets) contain various types of topics in our daily life, which include health-related topics. Analysis of health-related tweets would help us understand health conditions and concerns encountered in our daily lives. In this paper we evaluate an approach to extracting causalities from tweets using natural language processing (NLP) techniques.

**Methods:**

Lexico-syntactic patterns based on dependency parser outputs are used for causality extraction. We focused on three health-related topics: “stress”, “insomnia”, and “headache.” A large dataset consisting of 24 million tweets are used.

**Results:**

The results show the proposed approach achieved an average precision between 74.59 to 92.27% in comparisons with human annotations.

**Conclusions:**

Manual analysis on extracted causalities in tweets reveals interesting findings about expressions on health-related topic posted by Twitter users.

## Background

Twitter messages (tweets) have been a unique public resource for monitoring health-related information, including, but not limited to, disease outbreaks [1–3], suicidal ideation [4, 5], obesity [6], and sleep issues [7, 8]. Tweets provide diverse types of information about Twitter users, such as users’ behaviors, lifestyles, thoughts, and experiences. Automated causality extraction from tweets can help gather unique health-related information complementary to that from other data sources, such as research literature and electronic medical records. Despite the importance, the topic has not been extensively studied yet. This exploratory study considered automated extraction of attributable causes of health problems and concerns. We investigated whether causes of a given health problem or concern can be extracted from Twitter messages using natural language processing (NLP) techniques. We specifically focused on three health-related topics: stress, insomnia, and headache.

Text mining from tweets poses various challenges [9–11]. One of the challenges in studying causal relations is to accurately identify a small fraction of relevant tweets from a large data collection. In addition, language constructs within tweets are often informal and can make identification of causal relationships difficult. In this study, therefore, we aimed at precise extraction of causal relationships that are explicitly stated within a sentence. For example, given a tweet “*Excessive over thinking leads to insomnia*”, our goal is to extract “excessive over thinking” as a cause of “insomnia.” To this end, we created a set of lexico-syntactic patterns to extract “cause” information for a given “effect”. Quantitative and qualitative analyses were performed on causal relationships extracted from 24 million tweets.

Cause-effect relation extraction has been actively studied in the NLP field. There are two main approaches used for the task: 1) pattern/rule-based methods and 2) machine learning-based methods [12–15]. For example, Khoo et al. applied a dependency parser to each sentence and searched for extraction patterns in an obtained dependency graph using graph pattern matching. They reported an accuracy of 68.1–76.3% in identifying causality mentions in the Medline abstracts [16]. Girju and Moldovan used lexico-syntactic patterns involving causation verbs to extract cause-effect relations using pattern matching. They reported an accuracy of 65.6% on a news article corpus [12]. Similarly, Cole et al. [15] used a syntactic parser to identify triples of subject, verb, and object and applied various rules to determine which of these triples represent causal relations. They reported a precision and recall of 94.44 and 61.82%, respectively, on a news article corpus. Ittoo and Bouma automatically extracted causal patterns from Wikipedia and reported a ﻿precision of 76.5% and a recall of 82.0% on domain-specific documents (customer complaint and engineers’ repair action on medical equipment) [17, 18]. Recently, machine learning approaches have also been used to tackle causality extraction tasks [19–21]. Gijru used decision trees (C4.5) trained on 6000 sentences to extract causal relations and reported a precision of 73.91% and recall of 88.69% on a test set of 1200 sentences in the newswire domain [22]. Similarly, Blanco et al. [23] used decision trees to classify whether or not a pattern correctly encodes a causation and reported an average F-score of 89.5% on 1068 instances (75% training and 25% test) on a general English text corpus. Other works used support vector machines (SVMs) and conditional random fields (CRFs) with lexical, syntactic and semantic features and reported F-scores ranging from 0.82 to 0.85 on general English text corpus [24, 25]. Although the performance measures reported for machine learning-based methods are high compared with the pattern/rule-based approach, model training requires a large amount of manually-annotated data and a new model needs to be trained when target domains are changed. A comprehensive survey on causal relation extraction in the general NLP domain can be found in Asghar et al. [13].

Social media in general and Twitter in particular have been found as a useful and impactful resource in health-related surveillance studies. Twitter data have been used to mine topics related to depression [26, 27], mental health [28], stress and relaxation [29], and tobacco use [30]. Most common techniques for Twitter mining in the health-related domains are keyword/dictionary look-up and machine learning. Among the machine learning algorithms applicable to the tasks, support vector machines (SVM), logistic regression, and neural networks have been commonly used [1, 3, 29–31]. Although there have been Twitter studies in the health domain that concern causal relationships, such as the study of adverse reactions caused by drugs [32, 33] or various factors causing stress and relaxation [29], their focus is on a specific application and they do not investigate causal relation extraction itself. We believe there is a lack of studies on causality extraction from tweet in the health domain. To our best knowledge, this is the first study focusing on this problem.

## Methods

### Dataset

We used a corpus of 24 million tweets, collected from four cities (New York, Los Angeles, San Francisco and San Diego) over 4-month period (Sep 30, 2013 and Feb 10, 2014). Twitter Streaming API was used to retrieve 1% of all the tweets from these cities during the time period. This corpus was previously used to study stress and relaxation tweets [29]. Three terms: *stress*, *insomnia*, and *headache,* were selected as the target “effects”.

### NLP pipeline

**Fig. 1:**
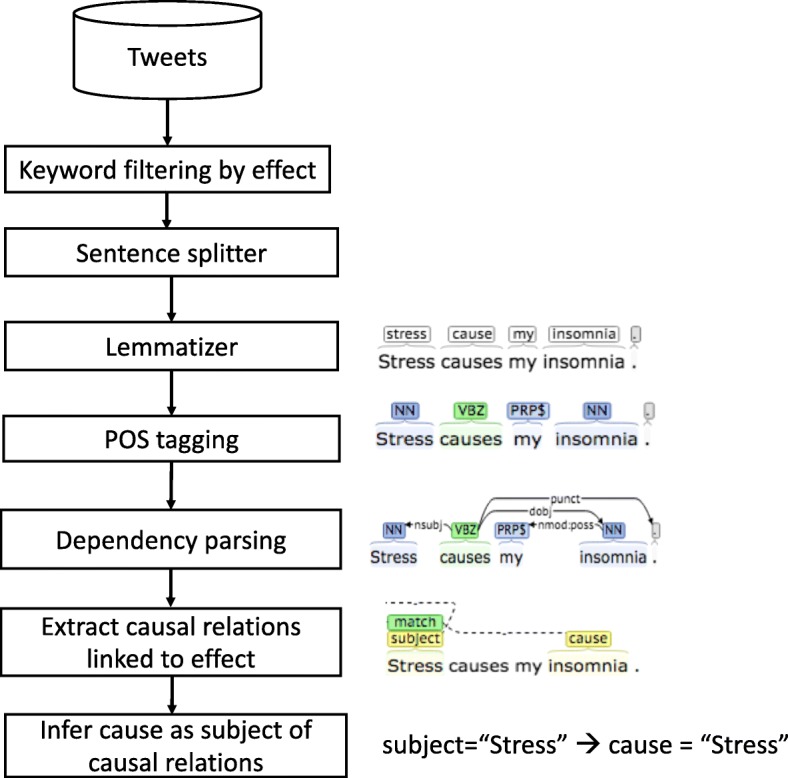
A general framework for causality extraction from Twitter messages

We aimed to develop a general method to extract causalities that is readily applicable to a new extraction target. We adopted an NLP framework that can leverage syntactic information to extract causal relations using a pattern/rule-based method. The NLP pipeline for this task is shown in Fig. 1. First, the corpus was filtered using the target keywords. Next, a series of basic NLP components were applied: sentence splitter, lemmatizer, Part-of-Speech (POS) tagger, and a dependency parser. Finally, causal relations were identified based on syntactic relations generated by the dependency parser. We used the CoreNLP package [34] (release version 3.8), a widely used Java library providing various NLP functionalities. The default settings and pre-trained models in the package were used for sentence splitter, lemmatizer, and POS tagger. For the parser, we selected the Probabilistic Context-Free Grammar (PCFG) parser and the pre-trained English model in the package, which generates a constituent tree for an input sentence and then converts it into a dependency graph. A dependency graph consists of vertices representing tokens (words and punctuations) and edges representing dependency relations among tokens [35]. Dependency relations are convenient for the purpose of extracting term relations in a sentence. Specifically, we used “Universal Dependencies” [35], which has a general annotation scheme to support multi-lingual and has been widely used in NLP community [35, 36]. Among the several options provided for dependency graph generation in the CoreNLP package, the method generateEnhancedDependencies that produces enhanced dependencies was used to derive dependency graphs from parsed trees.

### Cause-effect relation extraction

There are many different ways to state cause and effect relations, including verb phrases and noun phrases. As a result, we created rule set templates including trigger verbs and nouns. For example, a tweet containing “A caused B” has “caused” as a trigger verb and similarly “A result in B” has “result (in)” as a trigger verb. We initially created a list of trigger verbs and nouns by searching synonyms of “cause”, “result” and “reason” in WordNet (version 3.1), a widely used lexical database [37]. Ambiguous synonyms, such as “do” and “get”, were removed from the list. In the end, we selected seven verbs: “cause”, “stimulate”, “make”, “derive”, “trigger”, “result”, and “lead.” Similarly, we selected three nouns: “result”, “reason”, and “cause.”

The cause-effect relations were determined based on trigger terms as below:

*Trigger verb (active)*: “A <trigger> B”, where “<trigger>” is one of the seven selected verbs, e.g., “Stress *caused* insomnia”.

*Trigger verb (phrasal, active)*: “A <trigger> <preposition> B”, where <trigger> is “cause”, “result”, or “reason” and < preposition> is “in”, “to”, or “from”. E.g., “Stress *results in* insomnia”.

*Trigger verb (passive)*: “A <be> <trigger> by B”, where <be> is an appropriate form of be-verb, such as “is”, and < trigger> is a past participle form of a trigger verb. E.g. “Stress *was caused by* insomnia”.

*Trigger nouns*: “A <be> <trigger> of B”, where <trigger> is “cause”, “result”, or “reason” with an appropriate determiner. E.g., “Insomnia *is a result of* stress”.

Based on this principle, we created a set of six patterns to identify cause-effect relationship. To identify these patterns in a dependency graph derived from a sentence, we used CoreNLP Semgrex [38], which facilitates subgraph pattern matching over a dependency graph. The details of rules and their examples are listed in Table 1. For example, Rule 1 in Table 1 “{}=subj <subj ({word: /cause/}=target >dobj {}=cause)” indicates that the rule will match a sentence, such as “Stress caused my insomnia”, where “Stress” is matched with the pattern “{} = subj” and “insomnia” is matched with the pattern “{} = cause.” (Fig. 1). The base form of the trigger verbs and nouns could be used in these rules as the words were lemmatized by the Lemmatizer component of the CoreNLP library; for example, the base form “cause” is used in the rule to represent “causes”, “caused” and “causing”.

**Table 1:** Rule set to extract causal relations from tweets

#	Causal relation types	Dependency rules	Examples
1	A (noun) caused B	{} = subj < subj ({+ Causal verb +} = target >dobj {} = cause)	Stress causes insomnia
2	A (verb-ing) caused B	{} = subj < csubj ({+ Causal verb +} = target >dobj {} = cause)	Over thinking can increase anxiety and cause insomnia.
3	B was caused by A	{} = ncsubjpass<nsubjpass({+ Causal verb +} = target >/nmod:agent/{} = cause)	My insomnia was caused by stress.
4	A is a reason of B	Causal noun + < nsubj ({} = target > /nmod:of/ {} = cause)	Stress is a reason of my insomnia
5	B was caused by A (verb-ing)	{} = nsubj< nsubjpass ({} = target >/advcl:by/ + Causal noun)	Insomnia was caused by overthinking
6	A results “in/to/from” B	Causal verb + < [nc] subj ({} = target> /nmod:(to|in|from)/{} = cause)	Stress results to insomnia.

The final step was to extract causalities from identified cause-effect relations. We extracted the triple <cause, trigger, effect>, where effect is one of the three health-related topics of our focus: insomnia, stress, and headache.

## Results

We observed that the number of tweets containing specific health-related cause-effect relationships is small despite the large size of the Twitter corpus used. Specifically, the number of matched sentences was 501 out of 29,705 for stress (1.6%), 72 out of 3827 for insomnia (1.8%), and 94 out of 11,252 for headache (0.8%). The final causalities extracted were 41 for insomnia, 98 for stress, and 42 for headache. The details of the matching rules and the number of extracted causalities are shown in Table 2.

**Table 2:** Results when applying rule set in table i to a corpus of 24 millions tweets. The last rows indicates the numbers of tweets extracted with given effects (insomnia, stress and headache)

Matched rule #	Insomnia (of 3827)	Stress (of 29,705)	Headache (of 11,252)
1	58	381	78
2	4	12	3
3	0	4	1
4	1	21	2
5	0	32	0
6	9	51	10
Total	72	501	94
# extracted causalities	41	98	42

Table 2 also indicates that the majority of the relations were extracted by the pattern “A cause B” (Rule 1), followed by the pattern ‘A result in/to/from B’ (Rule 6). The rest of rules were used much less. This may suggest that Twitter users generally prefer direct and concise expressions. Notably, similar or the same phrases are repeated in collected tweets. For example, similar phrases *“missing someone causes insomnia”*, *“missing someone often causes insomnia”,* and *“missing someone causes insomnia like symptoms*” were found.

### Quantitative analysis

As stated, we aimed at precise extraction of causal relation using syntactic information. Therefore, we used the precision to evaluate our proposed method in this study, i.e., we manually reviewed tweets that were selected by the proposed method, instead of reviewing the entire Twitter corpus. The precision is calculated as the number of positive instances annotated by human annotators divided by the number of tweets the system found. The micro-average precision was calculated as the sum of all positives instances across all three categories divided by the sum of tweets the system found. Three human annotators [SD, EY, MT] discussed the annotation criteria and manually annotated the system outputs. We considered two annotation criteria: strict annotation and relaxed annotation. With the strict annotation, extracted relations were considered correct only when the cause of the target effect is clearly and explicitly stated. In the relaxed annotation, negated or hypothetical statements were additionally considered as correct extraction. For example, *“Cell phone radiation can cause insomnia”*, where the statement is hypothetical, was annotated as a false positive case in strict annotation, but a true positive case in relaxation. The disagreement in annotation were resolved by discussions among the annotators.

Table 3 shows the precision when comparing system outputs to human annotations. It shows that the micro-average for strict and relaxation is 74.59 and 92.27%, respectively. It also indicates that finding causal relationships for “headache” is more difficult than “insomnia” and “stress”. The large variations between the strict and relaxation evaluation (74.59% vs. 92.27%) also indicates that hypothetical assertions and negation play important roles in determining causal relationships in Twitter messages.

**Table 3:** Precision of extracted causalities when comparing to human annotators

	Strict evaluation	Relax evaluation (exclude hypothetical assertions and negation)
Insomnia	73.81%	88.10%
Stress	82.65%	96.94%
Headache	56.10%	85.37%
Micro-average	74.59%	92.27%

### Qualitative analysis

We further manually analyzed the causes of insomnia, stress and headache extracted by the system. Below are several findings.

#### Insomnia

We found that the most frequent cause related to insomnia was “missing someone”. Other causes include overthinking, social media (Facebook, Twitter), and hunger. Below are some examples of tweets and matching rules extracted from this topic:

- Missing someone causes insomnia.
- RULE 1: someone/NN...causes/VBZ ...insomnia/NN
- Night before first day of school always results in insomnia.
- RULE 6: Night/NN...results/VBZ ...insomnia/NN

#### Stress

Frequent topics related to stress for Twitter users include school, money, emails, computer games, and physical pains. Below are some of examples and matching rules for this topic.

- Money only causes stress and conflict
- RULE 1: Money/NN...causes/VBZ ...stress/NN
- School is the main cause of my stress
- RULE 4: School/NNP...cause/NN ...stress/NN

#### Headache

We observed the causes of headache reported in tweets include people, stress, crying, and listening. Below are some examples:

- My neck just made my headache 100x worse
- RULE 1: neck/NN...made/VBD ...headache/NN
- Nervous Stressed Leads to swollen eye & headaches
- RULE 6: Nervous/JJ...Leads/VBZ ...headaches/NNS
- You're the cause of my headaches.
- RULE 4: You/PRP...cause/NN ...headaches/NNS
- too many tears leads to headaches and heavy hearts
- RULE 6: tears/NNS...leads/VBZ ...headaches/NNS

Error analysis showed that false positive errors are often caused by complex sentences, such as *“Keeping to myself makes life way more stress free”,* or interrogative sentences, such as *“Could this be the cause of my insomnia?”*. Some false positive cases were due to sentences that do not explicitly indicate causality, such as *“I wouldn’t say it causes insomnia though”*. In such sentences, there are dependency links between subjects and given effects, and they match a rule in the extraction rule set. Including extraction rules that are more restricted and conducting more semantic analysis after extraction should further improve the precision.

## Discussion

Identifying target tweets precisely and efficiently is a primary concern in mining Twitter messages, which contains very large data and time-sensitive information. The goal in our experiment was to correctly identify tweets referring to causal information in a large data set using NLP. A dependency parser and associated NLP techniques were used to help improve precise information extraction. We also manually reviewed tweets identified by the proposed approach. We observed that the number of extracted causal relations is small. However, evaluation showed that it achieved a high precision. This indicates that using lexicon-syntactic relations derived from dependency parser yields high precision, which is an important factor in mining information from a large data set.

We further applied the framework to in-house clinical text since the proposed approach is general and flexible to customize. With modifications in our existing rules, we can correctly detect, within the clinical text, the association of a disease or symptoms with the patient. For example, the rule “{}=subj < nsubj ({lemma:/have/}=target >/dobj/ {}=cause)” can correctly assign assertions of disease not associated to patient such as “Parkinson’s” disease in the sentence *“Patient says his roommate has Parkinson’s and is concerned about his diagnosis”* or the second mention of “asthma” in *“Patient has no history of asthma, son has severe asthma”*. We believe it can benefit an NLP engine and has potential applications in the future.

## Limitations

The study has several limitations. First, the number of our rules and patterns are currently small and they may miss some expressions reporting cause-effect relations in sentences. Second, in this study we only considered a simple case of causal relation explicitly reported within one sentence. In reality, there can be cause-effect relation reported across sentences. Third, in Twitter messages, there are diverse ways in expressing cause-effect relations, including hashtags. The current approach that relies on syntactic patterns is unable to extract such information. Fourth, the data we used in this study is a small fraction (1%) from the real-world Twitter data. Although it was found that the fraction of tweets reporting causal relations was small, scalability of the method and the workflow is of important consideration in practice. Finally, we did not consider synonymous expressions of target concepts in the current study. It was beyond the scope of our exploratory study, but it must of great significance, especially when the synonymous expressions are not limited to noun phrases. e.g., “can’t sleep” as a synonymous expression of “insomnia.”

## Conclusions

In this paper, we presented an NLP approach to extracting causality from Twitter messages. The results on four months Twitter data revealed some interesting findings about different health-related topics. In the future we will focus more on semantic analysis, such as hashtags as well as multi-sentence causality extractions from tweets.

## Abbreviations

CRF: Conditional Random Field
NLP: Natural Language Processing
POS: Part-of-Speech
SVM: Support Vector Machine
